# Palonosetron: A novel approach to control postoperative nausea and vomiting in day care surgery

**DOI:** 10.4103/1658-354X.76484

**Published:** 2011

**Authors:** Sukhminderjit Singh Bajwa, Sukhwinder Kaur Bajwa, Jasbir Kaur, Veenita Sharma, Amarjit Singh, Anita Singh, SPS Goraya, SS Parmar, Kamaljit Singh

**Affiliations:** *Department of Anaesthesiology and Intensive Care, Gian Sagar Medical College & Hospital, Ram Nagar, Banur, Punjab, India*; 1 *Department of Obstetrics & Gynaecology, Gian Sagar Medical College & Hospital, Ram Nagar, Banur, Punjab, India*; 2 *Department of Biochemistry and Central Laboratory Services, Gian Sagar Medical College & Hospital, Ram Nagar, Banur, Punjab, India*

**Keywords:** *Day care surgery*, *palonosetron*, *ondansetron*, *PONV*

## Abstract

**Background:**

Postoperative nausea and vomiting (PONV) is one of the complications which hamper the successful implementation of day care surgical procedure in spite of the availability of so many antiemetic drugs and regimens for its prevention. The aim was to compare the prophylactic effects of intravenously (IV) administered ondansetron and palonosetron on PONV prevention in patients undergoing laparoscopic gynecological surgery under general anesthesia.

**Methods:**

A prospective double-blind study comprised of 60 ASAI/II female patients between the age group of 25 and 40 years was carried out in the Departments of Anesthesiology and Obstetrics and Gynecology of our institute. Patients were randomly divided into two groups of 30 patients each in a double-blind manner. Group I received 8 mg of inj. ondansetron IV while group II received inj. palonosetron 0.075 mg IV 5 minutes before the induction of anesthesia. The need for rescue antiemetics, episodes of PONV and other side effects were observed for 6 hours in the postanesthesia care unit and thereafter complaints were received on phone after the discharge. At the end of study, results were compiled and statistical data was subjected to statistical analysis using Student two-tailed ‘t’ and *χ*^2^ test and value of *P*<0.05 was considered significant.

**Results:**

The demographical profile of the patients was comparable. Twenty and 13.33% of the patients in group I had nausea and vomiting episodes postoperatively as compared to 6.67% and 3.33%, respectively, in group II which was statistically significant (*P*<0.05). Twenty percent of the patients in group I experienced significant post-op headache as compared to 6.67% in group II. The mean rescue dose of antiemetic was significantly higher (10.6 mg) in the group I as compared to group II (6.4 mg) (*P*=0.036). The rest of parameters were comparable and statistically nonsignificant.

**Conclusions:**

Palonosetron is a comparatively better drug to prevent the PONV in patients undergoing day care surgical procedures as compared to ondansetron as it has got a prolonged duration of action and favorable side-effects profile.

## INTRODUCTION

The popularity of day care surgery is on the rise since the last two decades. This has been made possible by a variety of factors including development of preanesthetic out-patient departments (OPDs), administration of lesser emetogenic anesthetic techniques, availability of modern monitoring devices for recovery period and advent of newer drugs for the prophylaxis of postoperative nausea and vomiting (PONV). The advantage of returning to the homely environment on the same day of surgery drive people of almost all the age groups to prefer surgery on outpatient basis. Lesser occupancy of hospital beds, lower incidence of infection, lower cost-benefit ratio and early resumption of professional and social activities are among the other possible benefits of day care procedures.[1,2] Smooth delivery of day care surgical services requires a highly dedicated staff, appropriate health infrastructure, adequate resources, established procedural guidelines as well as fully co-operative patients and their attendants.[3] As a result of so many huge requirements for efficiently delivering the day care services, protocols and guidelines of developed nations cannot be uniformly applied to the patients of developing world. In spite of all the advancements in day care surgery, PONV still remains a ‘big little problem’.[4] PONV is considered one of the most unpleasant postoperative discomforts in day care surgery which has got an incidence of 30-40% in normal population undergoing general anesthesia, while the incidence touches a peak of 75-80% in certain high-risk groups.[5,6] The incidence of PONV in the beginning of 19th century was quite high and used to be around 70-80% with the use of ether. With the use of modern-day anesthesia practices, the incidence of PONV has come down by 50% especially with the use of nonopioid medications for pain relief.[7] There is a strong correlation between the amount of postoperative use of opioids for pain relief and incidence of PONV and in one clinical trial it was established that dose reduction of opioids to half in 24 hour period can reduce the incidence of PONV by 6%. The amount of opioid administration should be carefully titrated so as to have minimal effect on the effectiveness of antiemetic drugs.[8,9] The risk and incidence of PONV with postoperative opioids use further increases if the opioids are delivered by patient-controlled analgesia.[10] The incidence of PONV is 5% in infants, 25% below 5 years, 40-50% in the 5-15 age group and 20-40% in adults.[11] The incidence of PONV is surprisingly less in the smokers as compared to nonsmokers but on the other hand the incidence of PONV is more in the patients who have history of sickness after consumption of alcohol. Most studies have found that incidence of PONV increases 1.5-2.5 times in patients with nonsmoking history and the incidence increases from 1.8 to 3.1 times in patients with prior history of motion sickness or PONV or both.[12–14]

The complete knowledge about the risk factors responsible for PONV helps in designing the treatment regimens and interventions for its control. PONV poses a great challenge to the surgeon as well as anesthesiologist as it causes a great discomfort, delay in discharge, increased readmissions to hospital, pulmonary complications and a delayed resumption of daily chores. Throughout the world, great amount of resources, time, capital and dedicated efforts are spent to find a better alternative for prevention of this irritating disturbance.[15,16] There is hardly any antiemetic drug available, which is complete in itself in suppression of PONV and the present generation of drugs just projects the picture of a mirage as far as adequate control of PONV is concerned. Although numerous pharmacological agents, regimens and techniques have evolved from time to time, they have limited efficacy due to various side effects and cost considerations. Nonpharmacological techniques like acupressure, acupuncture, accustimulation, etc. have been given various trials but their success is limited.[17,18]

The advent of 5-HT3 antagonists in medical practice has provided a great relief to the physicians, oncologists and anesthesiologists.[19] These pharmacological agents are as effective as any other antiemetic drug but with a more safety and favorable side-effects profile as they lack the sedative, dysphoric and extra-pyramidal side effects of other commonly used antiemetics.[20] All the 5-HT3 antagonists like ondansetron, dolasetron, granisetron, azasetron, tropisetron and palonosetron have a favorable drug profile and a long duration of antiemetic action (4-48 hours). Ondansetron is being routinely used throughout the world, either alone or in combination with other drugs, for the prophylaxis of PONV in day care surgery mainly because of its lower cost. Among these agents, palonosetron has got a far higher receptor affinity and a much longer half-life which confer a prolonged duration of action.[21] The long duration of antiemetic effect is quite beneficial in preventing the problem of PONV in day care procedures. The extensive research in the prevention of PONV has established 0.075 mg as the minimum effective dose of palonosetron and the same has been approved by FDA for PONV prophylaxis.[22,23] Patient characteristics, type of surgical procedure, duration of anesthesia and surgery are few of the important determinants of day care surgery and the procedures lasting for more than an hour are considered unsuitable for such selection. Risk factors for PONV include younger age, female gender, history of PONV and motion sickness, anxiety, nonsmoking status, history of migraine, certain ethnicities, general anesthesia, nitrous oxide, decreased perioperative fluids administration, increased duration of surgery, type of surgery such as laparoscopy, ear surgery, strabismus surgery and certain postoperative factors like pain, opioid analgesics and hypotension.[24] Gynecological patients are usually young and healthy. Females in the poor and developing countries like India are committed to their domestic and professional work as well as to the care of their family members. As a result of these commitments and obligations, these patients like to resume their daily chores as soon as possible and for them day care procedures with minimal postoperative discomfort are the preferred choices. In patients undergoing bilateral laparoscopic tubal ligation, CO_2_ levels as well as manipulation of visceral tissues during laparoscopy are potent emetogenic stimuli.[12,25] The role of long acting 5-HT3 antagonist acquires a greater dimension in prevention of PONV when such patients are operated on day care basis.

Keeping in consideration, the benefits of day care procedures and the long duration of antiemetic effect of palonosetron, we carried out a study in our institute consisting of 60 female patients, selected randomly, who underwent bilateral laparoscopic tubal ligation to see the comparative efficacy of ondansetron and palonosetron in the prophylaxis of PONV.

## METHODS

The present study was approved by the ethical committee of the institution and a written consent was taken from the patients after explaining to them in detail about the implications of the anesthetic and the surgical procedure. The selection criteria comprised of 60 ASA I/II patients between the age of 25 and 40 years who underwent bilateral laparoscopic tubal ligation surgery. Only those patients were chosen for study that lived in vicinity of 10-15 km radii from the institute and having a communication (mobile and landline phones) and personal transportation modes. The patients with ASA III/IV status, psychiatric diseases, diabetes, history of drug abuse, duration of surgery more than 1 h, chronic obstructive pulmonary disease, previous history of motion sickness and PONV, patients in premenstrual phase and body mass index >35 were excluded from the study.

A consultant anesthesiologist assessed all the patients in the evening before the surgery for preanesthetic evaluation. Patients were given a written set of information and instructions on preoperative preparation and postoperative care by the gynecologist to be followed as well as side effects to be observed for during the next 3 days. Patients were admitted in the early morning on the day of surgery. They were prescribed premedication with tablet ranitidine 150 mg and alprazolam 0.25 mg a night before and 2 hours prior to the surgical procedure. After securing a good IV access in the pre-op room patients were shifted to operation theatre and monitoring devices for ECG, heart rate, oxygen saturation and end-tidal carbon dioxide were attached. Pre-loading was done with 10 ml/kg body weight of Ringer lactate solution. Patients were randomly divided into two groups in a double-blinded manner with the help of computer-generated codes. Group I received 8 mg ondansetron IV while group II received 0.075 mg inj. palonosetron IV 5 minutes before the anesthesia procedure. These syringes were prepared by an anesthesia technician who was given a written set of instructions and was unaware of the procedure in the operation room. Induction of anesthesia in both the groups was achieved with inj. propofol 2 mg/kg body weight, inj. fortwin 20 mg and inj. midazolam 1 mg. Inj. atracurium 0.5 mg/kg body weight was administered for muscle relaxation and to aid in intubation. Maintenance of anesthesia was done with Inj. propofol 2 mg/kg/hr and atracurium 0.1 mg/kg given as per requirement. Patients were mechanically ventilated and were given 50% oxygen in air and a low dose of halothane. Infusion of diclofenac sodium 75 mg was administered during the procedure along with injection ranitidine 40 mg. After the completion of laparoscopic procedure, patients were given inj. reversal (neostigmine 2.5 mg and 0.5 mg of glycopyrrolate) and after thoroughly doing the oral suction, patients were extubated in a fully awake state. Patients were assessed in the recovery room both by the anesthesiologist and the gynecologist at every 15 mins interval for the first hour and at 30 mins interval thereafter. Any other complications like pain, PONV, etc. was looked for and recorded. Patients were discharged from the recovery room only after they fulfilled the following criteria.

- Tolerating oral plain fluids
- Patients were able to go to toilet by themselves
- Able to pass urine spontaneously
- Subjective feeling of betterment and a feel that they can manage themselves
- Presence of an adult person to accompany them.

Instructions written on the paper were reminded to them again verbally. Patients of both the groups were prescribed tab ondansetron 4 mg with a maximum dose of 12 mg/day as rescue antiemetic in case of any episode of nausea and vomiting at home. Oral diclofenac sodium was prescribed two times a day as rescue analgesic along with tablet ranitidine three times daily for the next 2 days. During the follow-up most of these patients recalled the postsurgical period as satisfactory and pleasant barring few complaints by small number of patients. At the end of the study all the data was systematically compiled and was subjected to Student two-tailed ‘t’ test and *χ*^2^ test. Value of *P*<0.05 was considered as significant.

## RESULTS

From January 2010 to May 2010, a total of 60 patients were evaluated randomly for day care laparoscopic tubal ligation. Results were prepared after questioning the patients during follow-up of postoperative period.

The demographic profile of the patients for the present study revealed no significant comparative difference between the two groups with respect to age, body weight, ASA grading, parity status and family income (*P*>0.05). However, the mean dose of recue antiemetic consumption was higher in the ondansetron group (10.6 mg) in the first 24 hours as compared to palonosetron group (64 mg) which on statistical comparison proved to be a significant entity (*P*=0.036) [Table 1]. Mean duration of surgery as well as mean duration of anesthesia was comparable in both the groups and on statistical analysis revealed no significant difference (*P*>0.05) [Table 1]. The number of patients who had episodes of nausea in immediate postoperative period were one in first one hour, another one in 1-6 hour period, two patients in period between 6 and 12 hours, one new patient in 12-24 hours and only one patient had episode of nausea after 24 hours in group I. In comparison, group II had one patient who suffered form nausea in the 1-6-hour period and another one in 6-12-hour period. Similarly, it was observed that four patients from group I and only 1 patient from group II had vomiting episodes in the first 24-hour period. The statistical values were significant in comparison (*P*<0.05) especially in the 6-12-hour period between both the groups [Table 2].

**Table 1:** Demographical profile of the patients of both the groups

Patient characteristic/Variable	Group I (n=30)	Group II (n=30)	*P*
Age (Mean ± SD)	33.46±1.86	32.22±1.58	0.76
Body weight	54.32±3.66	55.12±3.14	0.68
ASA grade I/II	21/9	19/11	0.42
Parity status:1/2/2+	6/21/3	4/22/4	0.36
Family income/month (in Rs)	<5000-3	<5000-5	0.29
	>5000-27	>5000-25	
Mean duration of surgery (in minutes)	27.86±4.68	29.24±3.88	0.84
Mean duration of anesthesia (in minutes)	36.42±2.58	38.26±2.96	0.63
Rescue dose of antiemetic (Ondansetron)	10.6*mg	6.4mg	0.036

* *P*<0.05 values are mean ± SD

**Table 2:** Comparison of incidences of nausea and vomiting in both the groups

Post-op duration (in hours)	Group I (n=30)		Group II (n=30)	
	Nausea	Vomiting	Nausea	Vomiting
0-1	1	1	0	0
1-6	1	1	1	0
6-12	2*	2*	1	0
12-24	1	0	0	1
24-72	1	0	0	0
Total	6 (20%)	4 (13.33%)	2 (6.67%)	1 (3.33%)

* *P*<0.05

It is very clearly evident that incidence of side effects are comparatively much lower in group II. The incidence of postoperative headache was significantly higher in the group I (*P*<0.05). The incidence of other side effects like pain, anxiety dizziness, constipation and myalgia were comparable and on statistical analysis revealed no significant difference (*P*>0.05) [Table 3].

**Table 3:** Comparison of incidences of side effects in both the groups

Side effects	Group I (n=30) No. of patients (%)	Group II (n=30) No. of patients (%)
Pain	2 (6.67)	3 (10)
Anxiety	2 (6.67)	1 (3.33)
Headache	6* (20)	2 (6.67)
Dizziness	2 (6.67)	1 (3.33)
Dry mouth	0	1 (3.33)
Sedation	1 (3.33)	0
Constipation	2 (6.67)	1 (3.33)
Myalgia	2 (6.67)	0

* *P*<0.05

## DISCUSSION

Day care surgery has proven over the years as the best method to reduce the burden on the health care resources as well as achievement of extreme patient satisfaction.[26] In developing countries like India, per capita income is much lower and most of the patients avoid bearing expenses of the prolonged hospital stay. In contrast to that, the health infrastructure in our country is not organized uniformly to smoothly deliver the day care procedures. Consequently the international guidelines formulated for such outpatient procedures are not easy to implement completely in poor and developing nations as compared to countries of developed world. In the present day scenario, PONV still remains a big headache and nuisance for the surgeons and anesthesiologists as well as an irritating discomfort for the patients almost equal in intensity to pain.[15,27] The delayed convalescence, hospital readmission, delayed return to work of ambulatory patients; postoperative surgical morbidities such as pulmonary aspiration, wound dehiscence, bleeding from the wound and metabolic derangement due to excessive emetic episodes are few of the adverse consequences of the PONV.[24] To compound the problems further, the lack of reliable and efficient transport, poor education level, lack of awareness, poorly developed referral system; underdeveloped communication systems, partially functioning primary healthcare services, and absence of community nursing have prevented the successful introduction of major surgical operations as day care surgery.

It is very difficult to predict the outcome in an individual patient as various other causes, besides the established risk factors, can influence the incidence of PONV. Various drugs regimens and antiemetic interventions have been tried fro time-to-time for prevention of PONV but with a variable success rate. The present study was carried out mainly to see the comparative efficacy of the new and much promising long-acting 5-HT3 antagonist palonosetron against ondansetron in day care surgery. The duration of surgery and anesthesia, type of surgery, young female patients, increased postoperative opioid requirements, etc., are few of the extensively studied risk factors.[12,25] The demographic profile of our patients was quite similar with majority of other research investigations and provided us the uniform platform to evenly compare the results obtained.[27,28] The mean duration of anesthesia and surgery were almost comparable in both the groups with no significant statistical difference. A consultant of anesthesia and gynecology were always present during the performance of these studies so as to confer uniformity to the study design as well to shorten the time of procedure which is quite similar to the profile of other researches.[12,25] The consumption of rescue antiemetics in our study was significantly higher in the ondansetron group particularly in the first 24 hours which can be due to the weaning of antiemetic effect of IV ondansetron as the effect lasts for 4-5 hour.

The prophylaxis for PONV is always on the mind whenever patients are selected for the surgery on day care basis. The purpose of day care surgery itself gets defeated if any such discomfort is faced by the patient after discharge from the hospital. The premenstrual phases in younger females who undergo laparoscopic surgery under general anesthesia for ambulatory gynecological surgery are very important risk factors for PONV.[12,24] But recently the premenstrual phase as a risk factor for PONV has been disapproved by many studies.[29]

As total IV anesthesia reduces the risk of PONV to quite an extent, we attempted to minimize the possible incidence of emetic episodes by using a propofol-based IV anesthesia and IV hydration as well as avoiding nitrous oxide.[30]

The overall incidence of nausea and vomiting was significantly higher in the ondansetron group as 20% and 13.33% of the patients experienced the nausea and vomiting episodes as compared to 6.67% and 3.33% in the palonosetron group, respectively. There were some patients who had multiple episodes of nausea and vomiting especially in the first 24 hours in both the groups but for comparison sake we included only the number of patients and not the number of episodes. The values were significant in comparison during the 6-12-hour period which again goes in accordance with the shorter duration of action of ondansetron. Rescue antiemetics were used by these patients during every episode of nausea or vomiting. Our results in the first 24-hour period with palonosetron are quite comparable with many other studies but the comparative efficacy against ondansetron has not been demonstrated by any literary evidence and only placebo has been used for comparison in various clinical trials.[23,31–34]

On statistical analysis, the observed power of the study was estimated at 0.584. These results have proved the better effectiveness of long acting palonosetron in the control of PONV as compared to ondansetron, thus providing the patients with a smooth and uneventful postoperative convalescent period and a lesser need for rescue antiemetics. The 5-HT3 antagonists exerts their antiemetic action by blocking the binding of serotonin to 5-HT3 receptors in the gut and the CTZ of area postrema which has got projections to the vomiting centre of lateral reticular formation of medulla oblongata.[35,36] The side-effect profile of palonosetron seems to be more favorable as only 6.67% of patients experienced post-op headache in our study as compared 20% of such incidence in ondansetron group. The established incidence of headache with palonosetron has been cited at approximately 9-12% in the literature.[34] The other side effects like dizziness, anxiety, constipation and myalgia have presented a nonsignificant picture and have shown a slightly higher incidence of these side effects in ondansetron group which is statistically insignificant. Ondansetron was the first member of this group to be marketed,[37] and the recommended IV dose (6-8 mg) is most effective when administered just after the completion of surgery because of its short duration of effect. Its antiemetic effect is stronger than its antinausea effect.[38] Both ondansetron and palonosetron have similar antiemetic efficacy but dose of palonosetron is much less than ondansetron. Intravenous dose of 0.075 mg of palonosetron is equivalent to 6-8 mg of IV ondansetron.[39] Moreover ondansetron has a shorter half-life of 3-5 hours, whereas palonosetron has a half-life of approximately 40 hours, which makes it more effective in preventing nausea and vomiting for day care surgery.[40] Majority of our results particularly the ones related to the incidence of nausea and vomiting may have been due to the difference in the duration of these two drugs. How the efficacy of different 5-HT3 receptor antagonists vary is still unclear but most probably these differences may involve multiple factors such as intrinsic differences in 5-HT3 receptor blocking activity, 5- HT3 receptor affinity and binding stability, and differences in autocrine activity of serotonin released from enterochromaffin cells to act on 5-HT3 or 5-HT4 receptors on EC cells.[41]

In conclusion, the results of the present study clearly conveys the facts that palonosetron has got a prolonged duration of antiemetic effect, a lesser need for rescue antiemetic postoperatively and a favorable side-effects profile as compared to ondansetron, thus providing the patients with smooth convalescent period with lesser nausea and emetic episodes who undergo laparoscopic gynecological surgery under general anesthesia.
